# Left bundle branch pacing and cardiac remodeling in HF patients with type 2 diabetes mellitus: epigenetic pathways and clinical outcomes

**DOI:** 10.3389/fphar.2024.1402782

**Published:** 2024-05-21

**Authors:** Celestino Sardu, Ludovica Vittoria Marfella, Valerio Giordano, Caterina Claudia Lepre, Giovanbattista D’Amico, Mario Volpicelli, Carla Contaldi, Raffaele Galiero, Alfredo Caturano, Flavia Casolaro, Ferdinando Carlo Sasso, Carlo Uran, Domenico Cozzolino, Maddalena Nicoletti, Giuseppe Signoriello, Giuseppe Paolisso, Raffaele Marfella

**Affiliations:** ^1^ Department of Advanced Medical and Surgical Sciences, University of Campania “Luigi Vanvitelli”, Naples, Italy; ^2^ Department of Cardiovascular Disease, “Vallo Della Lucania” Hospital, Salerno, Italy; ^3^ Department of Experimental Medicine, University of Campania “Luigi Vanvitelli”, Naples, Italy; ^4^ School of Geriatrics, University of L’Aquila, L’Aquila, Italy; ^5^ Cardiovascular Department, Santa Maria Delle Grazie Hospital, Nola, Italy; ^6^ Heart Failure Unit, Monaldi Hospital, Naples, Italy; ^7^ Department of Cardiovascular Diseases, San Giuseppe e Melorio Hospital, Santa Maria Capua Vetere, Italy; ^8^ UniCAMILLUS International Medical University, Rome, Italy

**Keywords:** heart failure, cardiac remodeling, reduced EF, T2DM, CRTd

## Abstract

**Background:**

Left bundle branch (LBB) pacing could achieve cardiac resynchronization therapy (CRT) in patients who cannot be resynchronized via the placement of the left ventricle (LV) lead into the coronary sinus. LBB pacing could improve cardiovascular outcomes in heart failure (HF) patients with LBB block who are affected by type 2 diabetes mellitus (T2DM).

**Study hypothesis:**

LBB pacing could increase the number of CRT responders and lead to the best clinical outcomes in HF patients with T2DM, inducing cardiac remodeling and improving left ventricle ejection fraction (LVEF) via microRNA (miR) modulation.

**Methods:**

In a multicenter observational study, we enrolled 334 HF patients with LBB block and an indication to receive LBB pacing for CRT. In these patients, we evaluated the CRT responder rate, clinical outcomes, and miR expression at 1 year of follow-up.

**Results:**

At 1 year of follow-up, we had 223 responders (66.8%), 132 hospitalizations for HF (39.5%), 24 cardiac deaths (7.2%), and 37 all-cause deaths (11.1%), with a higher rate of HF hospitalizations (77 (69.4%) vs 55 (24.7%), *p* < 0.05), and cardiac deaths (13 (11.7% vs 11 (4.9%), *p* < 0.05) in non-responders vs responders. At the end of follow-up, we found the lowest expression of miR-26, miR-29, miR-30, miR-92, and miR-145 in LBB-pacing non-responders vs responders (*p* < 0.05), and a direct correlation between miR-30 (0.340, [0.833–1.915]; p 0.001), the 6-minute-walking test (6MWT; 0.168, [0.008–0.060]; p 0.011), angiotensin-receptor-neprilysin inhibitors (ARNI; 0.157, [0.183–4.877]; p 0.035), sodium-glucose-transporter-2 inhibitors (0.245, [2.242–7.283]; p 0.001), and LVEF improvements. C reactive protein (CRP) inversely correlated with LVEF improvement (−0.220, [-(0.066–0.263)]; p 0.001). ARNI (1.373, CI 95% [1.007–1.872], p 0.045), miR-30 (2.713, CI 95% [1.543–4.769], p 0.001), and 6MWT (1.288, CI 95% [1.084–1.998], p 0.001) were predictors of LBB pacing responders at 1 year of follow-up.

**Conclusion:**

LBB-pacing responders evidenced miR modulation, which was linked to significant improvement of the cardiac pump. Specifically, miR-30 was linked to cardiac pump improvement and predicted responders at 1 year of follow-up in patients with T2DM.

## Introduction

Cardiac resynchronization therapy (CRT) is recommended for patients with heart failure (HF) and left bundle branch (LBB) block (Glikson et al., 2021). Indeed, CRT could restore ventricular synchrony and improve LV hemodynamics by left ventricular lead placement through the coronary sinus (CS) (Glikson et al., 2021). These effects could improve exercise tolerance, reducing HF hospitalizations and mortality via LV reverse cardiac remodeling (Sardu et al., 2018a; Glikson et al., 2021). LV reverse cardiac remodeling is linked to the reduction of cardiac hypertrophy, fibrosis, and apoptosis via the modulation of specific microRNAs (miRs) (Marfella et al., 2013; Sardu et al., 2016). These effects are seen in patients defined as CRTd responders and linked to the modulation of a few miRs, which are differently expressed in non-responder vs responder patients (Marfella et al., 2013; Sardu et al., 2016). Notably, a higher percentage of CRT patients have type 2 diabetes mellitus (T2DM), which could negatively affect the responder rate and clinical outcomes (Marfella et al., 2013; Sardu et al., 2016; Sardu et al., 2018a; Glikson et al., 2021). Conversely, about 5%–7% of selected patients cannot receive CRT because of unsuccessful or complicated LV lead placement through CS and evidence of other limiting factors (Vijayaraman et al., 2021). The authors proposed CRT via LBB pacing in this setting, which is feasible and safe and provides an alternative treatment (Vijayaraman et al., 2021). Intriguingly, LBB pacing could lead to cardiac remodeling and the best clinical outcomes in HF patients who cannot receive CRT via CS pacing (Chen et al., 2022). On the other hand, the benefits of LBB pacing on cardiac remodeling and clinical outcomes are still under-investigated at the molecular and epigenetic levels in HF patients with T2DM. Therefore, here we evaluated the effects of LBB pacing on CRT response (responder rate) as the primary study endpoint and the rates of all causes of death, cardiac deaths, and HF hospitalizations in patients with T2DM as secondary study endpoints. Then, we evaluated the miRs differently expressed in LBB-pacing non-responders vs responders with T2DM at baseline and at 1 year of follow-up. Finally, we evaluated the miRs and other study variables correlated with echocardiographic indexes of cardiac pump improvement (increase of LV ejection fraction, LVEF> 10%) at 1 year of follow-up in LBB-pacing patients with T2DM.

## Methods

In all the participating centers, we enrolled consecutive HF patients with LBB, diagnosed T2DM, and indicated to receive CRT via LBB pacing (Glikson et al., 2021). The T2DM diagnosis was made according to the diagnostic criteria of the American Diabetes Association (Ref Moghissi et al., 2009). These patients did not receive the CRT via the placement of a left ventricular lead through the coronary sinus because it was not possible to reach the optimal LV pacing site via the right branch of the coronary sinus or by evidence of occlusion of the coronary sinus anatomy, phrenic nerve stimulation, and other anatomical constraints negatively influencing the CRT procedure (Vijayaraman et al., 2021; Chen et al., 2022). The screened T2DM patients answered a specific questionnaire about medicines used for diabetes treatment, the date of the beginning and end of treatment, the route of administration, and the duration of use (Ref Moghissi et al., 2009). These patients received CRT via the LBB pacing and met the following inclusion and exclusion criteria:

Inclusion criteria: age 18–80 years, sinus rhythm, LBB block, diagnosis of well-controlled T2DM (glycated hemoglobin (Hb1Ac) ≤7%), and clinical history of stable chronic HF under at least 3 months of guideline-directed medical anti-HF therapy (Gorcsan et al., 2008); NYHA functional class II or III, severe left ventricle ejection fraction reduction (LVEF <35%), stable sinus rhythm and indication to receive CRT (Glikson et al., 2021). The patients who respected the criteria received a CRT implant for LBB pacing (Vijayaraman et al., 2021).

Exclusion criteria: non-LBB QRS morphology, diagnosis of right bundle branch block (RBBB) or intraventricular conduction delay; T2DM with Hb1Ac>7%, unstable HF, NYHA functional class IV, persistent atrial fibrillation; patients previously treated with CRT, pacemakers, or internal cardioverter defibrillator implants; hyperkalemia, systolic hypotension (systolic blood pressure <90 mmHg); patients with an estimated glomerular filtration rate (eGFR) of at least 30 mL per minute per 1.73 m^2^ of body surface area; pregnancy, inflammatory chronic systemic disease, or oncological disease; absence of informed patient consent, and any condition that would make survival for 1 year unlikely. The Institutional Review Boards at the enrolling hospitals approved the study, and all patients provided informed consent for the intervention (LBB-CRT pacing) and to participate in the study. Then, the enrolled study population was divided into two groups: non-responders vs responders to CRT via LBB pacing according to trans thoracic echocardiographic evidence of a significant cardiac pump increase and LV reverse remodeling and significant change in functional HF class (NYHA class amelioration).

### Study design

We performed a multicenter, observational study with a follow-up of 1 year. We performed LBB pacing on all the study patients for CRT. In this cohort of patients with HF and T2DM, we evaluated the CRT responder rate as the primary study endpoint and all causes of death, cardiac deaths, and HF hospitalizations at 1 year of follow-up as secondary study endpoints. Finally, we evaluated the miRs expressed at baseline and at the end of follow-up in the LBB-pacing non-responders vs responders. Then, we correlated the clinical variables and the investigated miRs to the echocardiographic parameters of a significant increase in cardiac pump at the end of follow-up.

### Anthropometric and echocardiographic evaluations

The authors performed a physical examination of all enrolled patients, with evaluation of vital signs and revision of adverse events at follow-up. In the study cohorts, we performed a transthoracic two-dimensional echocardiogram with M-mode recordings, conventional Doppler, and pulsed-wave tissue Doppler imaging (TDI) measurements at baseline and then at 12 months of follow-up using a Philips iE33 echocardiograph (Eindhoven, Netherlands). The left ventricle end-diastolic diameters (LVEDD), end-diastolic volumes (LVEDV), end-systolic diameters (LVESD), and end-systolic volumes (LVESV) were measured. We calculated the LVEF with the Simpson method (Gorcsan et al., 2008). We classified the grading of mitral regurgitation as low (+), moderate (++), moderate-severe (+++), and severe (++++) (Jankowska et al., 2011). The diagnostic exam was performed by physicians fully trained in echocardiography, blinded to the study protocol. Finally, the authors analyzed all echocardiographic data.

### Implanting procedures and device programming

Experienced physicians in LBB pacing performed the implantation using the LBB pacing catheters (SelectSecure, model 3,830, Medtronic Inc., Minneapolis, United States; Solia S, Biotronik, Berlin, Germany) and dedicated delivery sheaths (C304 and C 315, Medtronic Inc., Minneapolis, United States; Selectra 3D, Biotronik, Berlin, Germany) available for use (Sardu et al., 2016; Vijayaraman et al., 2021). The LBB pacing lead was introduced into the right ventricle (RV) and placed on the right side of the interventricular septum (IVS), and here advanced deeply into the IVS until it reached the LV septal subendocardium and the RBBB morphology of the paced QRS complex was observed in electrocardiogram (ECG) lead V1 (Vijayaraman et al., 2021; Chen et al., 2022). We performed a pacing test during the procedure. Surface 12-lead ECG, intracardiac electrograms, and fluoroscopy were simultaneously monitored. We measured the pacing stimulus-to-LV activation time (LVAT) in lead V5 or V6 at low (<3 V/0.5 ms) and high (>5 V/0.5 ms) outputs. The lead tip was considered to be at the final position once LBB capture was confirmed by evidence of 1) the LBBB morphology disappearance, with a paced RBBB pattern (typical or atypical) observed in lead V1; 2) LVAT<100 ms at low pacing output (<3 V/0.5 ms), (Vijayaraman et al., 2021; Chen et al., 2022). We assessed the penetration depth in the IVS by injecting contrast medium through the sheath in the left anterior oblique 45-degree view during the procedure and by transthoracic echocardiography before discharge. Then, we used the CRT with a defibrillator (CRTd) device, then connecting the LBBP lead to the LV port of the device (Vijayaraman et al., 2021; Chen et al., 2022). We programmed the VV delay with devices in DDD mode to ensure exclusive LBBP with atrioventricular (AV) delay optimization for the shortest QRS interval duration. At the end of the procedure, we confirmed the final position of the CRT leads by catheter interrogation and cine fluoroscopy view.

### Laboratory analysis

After an overnight fast in all patients, we evaluated the plasma glucose, HbA1c, serum lipids, and B-type natriuretic peptide (BNP) by enzymatic assays. We evaluated serum levels of pro-inflammatory cytokines (tumor necrosis factor-alpha (TNFα), interleukin-1, (IL-1), and interleukin-6 (IL-6)), systemic inflammatory markers (C reactive protein, CRP), leucocytes and neutrophils count at baseline and 12-month follow-up (Sardu et al., 2018b; Adamo et al., 2020). We used commercially available enzyme-linked immunosorbent assays (ELISAs) kits to determine the TNFα, IL-1, IL-6, and CRP (TNFα: TNF alpha Human ELISA Kit KHC3011, Thermo Fisher Scientific; IL-1: Hu-man IL-1 α ELISA Kit RAB0269, Sigma-Aldrich; IL-6: Human IL-6 Quantikine ELISA Kit D6050, R&D Systems; CRP: CRP Human ELISA kit KHA0031, Thermo Fisher Scientific). An ice-cooled blood collection system was used to collect blood samples, which were immediately centrifuged for 10 min at 2,500 rpm at 4°C. Before proceeding with ELISAs, we isolated the supernatants containing serum samples and then stored them at −80°C.

### RNA serum extraction and miR analysis

We extracted 200 µL of serum from each enrolled CRT patient via peripheral venous blood samples at baseline and follow-up. We used the miRNeasy Mini kit to characterize the miR expression (Qiagen, 20124 Milan, Italy) (Marfella et al., 2013; Sardu et al., 2016). A single reaction for RNA isolation was carried out by pooling eight serum samples extracted from patients matched for sex, age, and clinical evaluations. Then, we assayed the miRs from blood samples at baseline and quarterly during 12 months of follow-up in LBB-responders vs LBB-non-responder patients. We evaluated the miRs implied in various processes of HF and previously evaluated and differently expressed in CRT defibrillator (CRTd) responders vs non-responders (Marfella et al., 2013; Sardu et al., 2016). Thus, we spiked a 5-µL aliquot of 5 nM Syn-cel-miRNA-39 miScript miRNA-Mimic from the total RNA, including small RNAs, before nucleic acid preparation to monitor the efficiency of miR recovery and to normalize subsequent miR expression (Marfella et al., 2013; Sardu et al., 2016). We evaluated the serum expression of the miR-26b-5p, miR-29a-3p, miR-30e-5p, miR-92a-3p, and miR-145-5p, as previously seen in the CRT response (Marfella et al., 2013; Sardu et al., 2016). To date, we performed triplicate determinations of hsa-miR-26 (MIMAT0000083), miR-29 (MIMAT0000086), miR-30 (MIMAT0000692), miR-92 (MIMAT0000092), miR-145 (MIMAT0000437), and Ce_miR-39-3p (MIMAT0000010) through a CFX96 Real-Time System C1000 Touch Thermal Cycler (BioRad Laboratories, Inc), by using miScript SYBR Green PCR kit (218073, QIAGEN) and specific miScript primer assays (MS00003234, MS00003262, MS00009401, MS00006594, MS00003528, and MS00019789) (Lappegård et al., 2015; Vijayaraman et al., 2021; Chen et al., 2022). qRT-PCR data were analyzed by using the 2^-ΔΔCt^ method, where cycle threshold (Ct) values were determined by CFX Manager™ Software (BioRad Laboratories, Inc).

### Study endpoints

We evaluated the following primary and secondary study endpoints at 1 year of follow-up. Primary study endpoint: the rate of CRT responders to LBB pacing. We investigated the rate of CRT responders by the diagnosis of LV reverse remodeling (reduction in LVESV ≥10%) and significant increase of cardiac pump (assessment of LVEF ≥10%) assessed by transthoracic echocardiography, and significant change in functional HF class (improvement of the six min-walk test (6MWT) and Minnesota Living with HF scale improvement). Therefore, at follow-up, we re-evaluated the NYHA classification for each patient. The enrolled patients were instructed regularly to assess body weight, the occurrence of dyspnea, and any clinical symptoms. The patients graded their overall condition as unchanged or slightly, moderately, markedly worsened, or improved by global self-assessment (Sardu et al., 2018a).

-Secondary endpoints: all causes of death, cardiac deaths, and HF hospitalization events. Then, in these two groups of patients (responders vs non-responders), we selectively evaluated the miR expressions at baseline and 1 year of follow-up. Finally, we evaluated the miRs and study variables correlated to echocardiographic indexes of cardiac pump improvement (an increase of LVEF >10%) at 1 year of follow-up in LBB-pacing patients.

### Statistical methods

The data were collected and then analyzed by a qualified statistician. The HF patients treated by LBB pacing were then divided into non-responder vs responder cohorts. We performed the safety analyses on data from all HF and LBB-enrolled patients. Continuous variables were expressed as means and standard deviations and tested by a two-tailed Student’s t-test. The categorical variables were compared using the chi-square or Fisher exact test where appropriate. We calculated the number of patients who showed echocardiographic evidence of significant improvement in cardiac pump (increase of LVEF ≥10%) at 1 year of follow-up. We reported this study event as a “yes” (score 1) or “not” (score 0). Then, we evaluated by multiple regression analysis its relationship with miRs and other study variables (after the evaluation of the normal distribution of model variables by residue analysis) at the end of follow-up. Finally, the predictors of the response (responder rate, as primary study endpoint) to CRT via LBB pacing at 1 year of follow-up were evaluated using Cox regression models in the study population adjusted for study variables: age, angiotensin-receptors-neprilysin inhibitors (ARNI), beta-blockers, BMI >30 kg/m^2^, BNP, CRP, HbA1c, hypertension, LVEF, miR-30, miR-145, QRS at baseline, sodium-glucose-transporter 2 inhibitors (SGLT2i), II NYHA class, and 6MWT. A *p*-value < 0.05 was considered statistically significant. The statistical analysis was performed using the SPSS software package for Windows 17.0 (SPSS 23 Inc., Chicago, Illinois).

## Results

**FIGURE 1:**
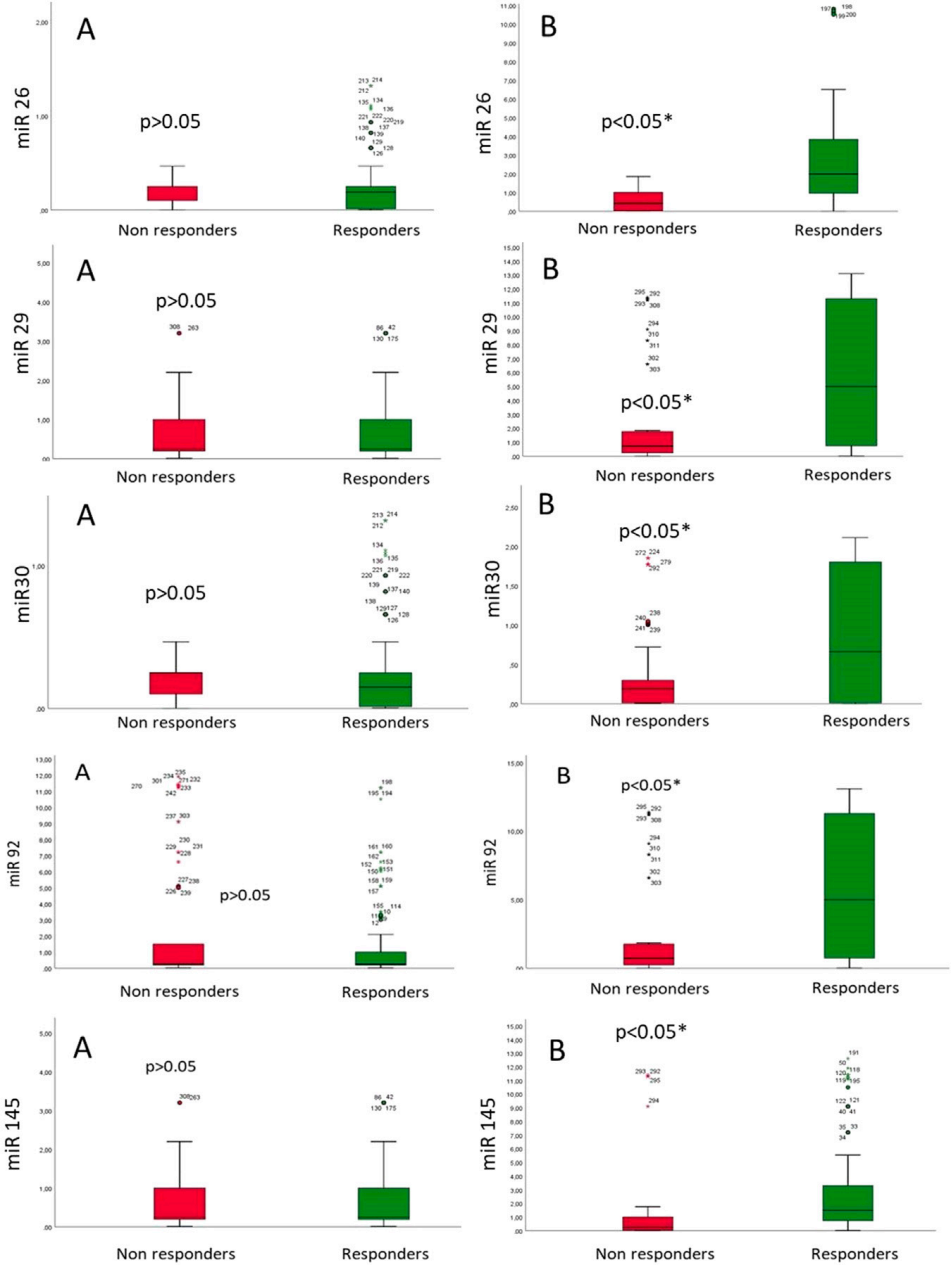
MicroRNA (miR) expression at baseline **(A)** and the end of follow-up **(B)** in patients with HF and T2DM in LBB-pacing non-responders vs. responders.

- Baseline findings: The characteristics of overall T2DM patients treated by LBB pacing (n = 334) and study cohorts divided as non-responders (n = 111) vs responders (n = 223) were reported in Table 1. At baseline, we did not find a significant difference between the LBB-pacing non-responders vs responders (p > 0.05) Table 1.
- Effects of LBB pacing on clinical parameters: At 1 year of follow-up, comparing LBB-pacing non-responders vs responders, we found significant differences regarding the clinical parameters of NYHA class (worsening of NYHA class), higher QRS duration, and BNP values, and lower values of 6MWT (p < 0.05); see Table 1. Non-responders showed higher values of inflammatory markers (p < 0.05) than responders; see Table 1. Conversely, non-responders showed the lowest values of LVEF (worsening of the cardiac pump) and highest values of end-diastolic and end-systolic diameters and volumes (p < 0.05); these study cohorts evidenced significant differences in the grade of mitral insufficiency (p < 0.05); see Table 1. Finally, at 1 year of follow-up, LBB-pacing non-responders vs responders showed a higher rate of patients receiving ARNI and SGLT2-I therapy (p < 0.05) Table 1.
- Primary and secondary study endpoints: at 1 year of follow-up, we reported 223 responder patients (66.8%) in the overall population, 132 hospitalizations for HF (39.5%), 24 cardiac deaths (7.2%), and 37 events of all-cause death (11.1%). We found a higher rate of HF hospitalizations (77 (69.4%) vs 55 (24.7%), p < 0.05) and cardiac deaths (13 (11.7%) vs 11 (4.9%), p < 0.05) in non-responders at 1 year of follow-up. The two groups did not differ in the rate of all causes of death (15 (13.5%) vs 22 (9.9%), p > 0.05) at 1 year of follow-up.
- MiR expression: In Figure 1, we reported the miR expression at baseline vs end of follow-up in LBB-pacing non-responders vs responders. At baseline (A part), we did not find significant differences in miR expression (miR-26, miR-29, miR-30, miR-92, and miR-145) in LBB-pacing non-responders vs responders; see Figure 1. At the end of follow-up (B part), we found the lowest expression of miR-26, miR-29, miR-30, miR-92, and miR-145 in the LBB-pacing non-responders vs responders (p < 0.05); see Figure 1.
- MiR changes and clinical parameters: The multiple variable regression analysis showed the relationship between study variables and cardiac pump (LVEF) improvement at 1 year follow-up post-LBB-pacing. Thus, we found a direct correlation between miR-30 (0.340, [0.833–1.915]; p0.001), 6MWT (0.168, [0.008–0.060]; p 0.011), ARNI (0.157, [0.183–4.877]; p 0.035), and SGLT2i (0.245, [2.242–7.283]; p 0.001). In contrast, we found an inverse correlation between CRP (−0.220, [-(0.066–0.263)]; p 0.001) and LVEF improvement. Then, we used Cox regression analysis and found that ARNI therapy (1.373, CI 95% [1.007–1.872], p 0.045), miR-30 (2.713, CI 95% [1.543–4.769], p 0.001), and 6MWT (1.288, CI 95% [1.084–1.998], p 0.001), were predictors of LBB-pacing responders at 1 year of follow-up; see Table 2.

**TABLE 1:** Clinical characteristics of our study population at baseline and at the end of follow-up as CRT non-responders vs responders.

		Baseline			1-year follow-up		
Clinical parameter	Overall LBB pacing patients (n = 334)	Non-responders (n = 111)	Responders (n = 223)	*p*-value	Non-responders (n = 111)	Responders (n = 223)	*p*-value
Age, years	70.7 ± 6.2	70.4 ± 5.9	70.8 ± 6.4	0.868	71.5 ± 5.9	71.8 ± 6.4	0.686
Male, n (%)	239 (71.5)	75 (67.6)	164 (73.5)	0.260	—	—	—
Smokers, n (%)	175 (52.4)	52 (46.8)	123 (55.2)	0.164	60 (54.1)	134 (60.1)	0.346
BMI >30 kg/m^2^ (%)	24 (7.2)	9 (8.1)	15 (6.7)	0.657	11 (9.9)	19 (8.5)	0.688
Hypertension, n (%)	237 (70.9)	80 (72.1)	157 (70.4)	0.799	84 (75.7)	162 (72.6)	0.599
Dyslipidemia, n (%)	136 (40.7)	49 (44.1)	87 (39.0)	0.408	45 (40.5)	80 (35.9)	0.472
Ischemic heart failure, n (%)	224 (67.1)	73 (65.8)	151 (67.7)	0.803	84 (75.7)	162 (72.6)	0.599
Plasma glucose (mg/dL)	147.1 ± 23.2	146.3 ± 24.1	148.9 ± 22.7	0.388	141.6 ± 24.1	140.8 ± 20.7	0.356
HbA1c (%)	6.8 ± 0.36	6.8 ± 0.32	6.7 ± 0.41	0.084	6.6 ± 0.38	6.5 ± 0.41	0.113
NYHA class, n (%)				0.402			0.001*
I NYHA class		—	—		5 (4.2)	22 (9.9)	
II NYHA class		25 (22.5)	56 (25.1)		29 (26.1)	99 (44.4)	
III NYHA class		86 (77.5)	167 (74.9)		69 (62.2)	96 (43.0)	
IV NYHA class		—	—		8 (7.2)	6 (2.7)	
QRS duration (ms)	136.5 ± 8.5	135.9 ± 8.7	136.7 ± 8.5	0.187	130.8 ± 12.2	127.7 ± 11.6	0.022*
6MWT	240.32 ± 39.6	195.86 ± 32.53	190.46 ± 26.12	0.407	218.17 ± 44.15	247.17 ± 44.52	0.013*
BNP (pg/mL)	420.73 [85.26–1,440.1]	398.87 [58.10–1,398.87]	464.66 [62.3–1,442.13]	0.090	234.98 [83.12–751.15]	157.98 [93.1–562.12]	0.001*
**Inflammatory biomarkers**							
Lymphocytes	7.47 ± 1.23	7.21 ± 1.08	7.65 ± 1.27	0.138	7.87 ± 1.68	7.30 ± 1.42	0.001*
Neutrophils	5.94 ± 0.98	5.84 ± 1.02	5.99 ± 1.02	0.186	5.83 ± 0.98	5.50 ± 1.24	0.001*
CRP (mg/L)	8.9 ± 0.98	8.66 ± 0.76	9.05 ± 0.51	0.734	6.29 ± 0.37	6.13 ± 0.40	0.001*
IL6 (pg/mL)	6.53 ± 0.06	6.54 ± 0.07	6.48 ± 0.03	0.162	6.30 ± 0.09	5.99 ± 0.82	0.001*
TNFα (pg/mL)	6.37 ± 0.04	6.35 ± 0.03	6.37 ± 0.04	0.744	6.36 ± 0.02	6.13 ± 0.02	0.001*
**Echocardiographic parameters**							
LVEF (%)	30.1 ± 5.2	30.7 ± 4.2	29.7 ± 5.5	0.114	34.2 ± 9.3	46.1 ± 5.5	0.001*
LVEDd (mm)	71.2 ± 3.9	71.5 ± 4.1	71.0 ± 3.8	0.276	69.5 ± 5.7	65.2 ± 3.8	0.001*
LVESd (mm)	39.7 ± 2.4	41.5 ± 3.8	39.6 ± 2.4	0.289	39.9 ± 2.6	38.6 ± 4.8	0.152
LVEDv (mL)	240.9 ± 27.9	241.5 ± 14.7	243.9 ± 16.8	0.193	228.4 ± 19.7	210.5 ± 14.1	0.001*
LVESv (mL)	131.3 ± 11.9	130.9 ± 19.3	133.3 ± 21.5	0.062	125.6 ± 15.8	104.9 ± 16.8	0.001*
Mitral insufficiency				0.812			0.046*
+ (%)		53 (47.7)	102 (45.7)		44 (39.6)	112 (50.2)	
++ (%)		41 (36.9)	89 (39.9)		45 (40.5)	98 (43.9)	
+++ (%)		17 (15.3)	32 (14.3)		18 (16.2)	13 (5.8)	
++++ (%)					3 (2.7)	—	
**Medications**							
Beta-blockers, n (%)	220 (65.9)	74 (66.7)	146 (65.5)	0.903	81 (73.0)	162 (72.6)	0.443
Carvedilol		56 (75.7)	105 (71.9)		60 (74.1)	120 (74.1)	
Bisoprolol		18 (24.3)	41 (28.1)		21 (25.9)	42 (25.9)	
Calcium antagonist, n (%)	16 (4.8)	5 (4.5)	11 (4.9)	0.863	4 (3.6)	9 (4.0)	0.940
Amiodarone, n (%)	63 (18.9)	20 (18.0)	43 (19.3)	0.882	27 (24.3)	48 (21.5)	0.443
ACE inhibitors, n (%)	84 (25.1)	25 (22.5)	59 (26.5)	0.504	32 (28.8)	60 (26.9)	0.795
ARS blockers, n (%)	87 (26.0)	33 (29.7)	54 (24.2)	0.292	35 (31.5)	60 (26.9)	0.440
ARNI, n (%)	101 (30.2)	29 (26.1)	72 (32.3)	0.258	52 (46.8)	78 (35.0)	0.043*
Aspirin, n (%)	124 (37.1)	43 (38.7)	81 (36.3)	0.719	48 (43.2)	84 (37.7)	0.344
Warfarin, n (%)	99 (29.6)	37 (33.3)	62 (27.8)	0.411	39 (35.1)	66 (29.6)	0.319
NOAC, n (%)	65 (19.5)	25 (22.5)	40 (17.9)	0.379	26 (23.4)	45 (20.2)	0.270
Ticlopidine, n (%)	8 (2.4)	2 (1.8)	6 (2.7)	0.724	4 (3.6)	6 (2.7)	0.736
Ivabradine, n (%)	103 (30.8)	33 (29.7)	70 (31.4)	0.802	31 (27.9)	70 (31.4)	0.530
Digoxin, n (%)	98 (29.3)	34 (30.6)	64 (28.7)	0.799	36 (32.4)	68 (30.5)	0.802
Diuretics, n (%): loop diuretics		89 (80.2)	188 (84.3)	0.357	101 (91.0)	196 (87.9)	0.462
Tiazides		13 (11.7)	24 (10.8)	0.517	14 (12.6)	31 (13.9)	0.865
Aldosterone blockers		67 (60.4)	129 (57.8)	0.559	87 (78.4)	155 (69.5)	0.093
Statins, n (%)	234 (70.1)	81 (73.0)	153 (68.6)	0.448	85 (76.6)	160 (71.7)	0.362
SGLT2-I, n (%)	71 (21.2)	24 (21.6)	47 (21.1)	0.909	38 (34.2)	50 (22.4)	0.041*
Anti-DM medications, n (%)				0.631			0.813
-oral hypoglycemic	295 (88.3)	96 (86.5)	199 (89.2)		99 (89.2)	202 (90.6)	
-insulin	71 (21.3)	25 (22.5)	46 (20.6)		28 (25.2)	53 (23.8)	
-DPP4i	68 (20.4)	24 (21.6)	44 (19.7)		25 (22.5)	52 (23.3)	
-GLP1-RA	53 (15.9)	17 (15.6)	36 (16.1)		20 (18.0)	39 (17.5)	

**TABLE 2:** Cox regression analysis for primary study endpoint (LBB-pacing responders) at 1 year of follow-up.

	Univariate analysis			Multivariate analysis		
Risk factor	HR	CI, 95%	*p*-value	HR	CI, 95%	*p*-value
Age	1.007	0.983–1.031	0.565			
ARNI	1.375	1.054–1.795	0.019*	1.373	1.007–1.872	0.045*
Beta-blockers	1.041	0.789–1.372	0.777			
BMI >30 kg/m^2^	0.748	0.443–1.263	0.277			
BNP	1.015	0.989–1.102	0.277			
CRP	1.002	0.987–1.016	0.808			
Glycemia	0.852	0.650–1.117	0.248			
Hb1Ac	0.101	−0.181–0.192	0.084			
Hypertension	1.053	0.789–1.404	0.726			
LVEF	0.950	0.847–0.973	0.047*	0.956	0.927–1.126	0.104
miR-30	1.954	1.109–3.441	0.020*	2.713	1.543–4.769	0.001*
miR-145	1.015	0.859–1.199	0.863			
QRS duration	1.013	0.998–1.028	0.097			
SGLT2i	0.837	0.606–1.154	0.277			
II NYHA class	1.080	0.825–1.413	0.576			
6MWT	1.192	1.018–1.996	0.001*	1.288	1.084–1.998	0.001*

## Discussion

In our study, 223 T2DM patients (66.8%) were found to respond to CRT via LBB-pacing at 1 year of follow-up. LBB-pacing non-responders vs responders showed a worse NYHA class, higher QRS duration and BNP values, and the lowest values of 6MWT at 1 year of follow-up (*p* < 0.05). This negative clinical trend is linked to over-inflammation, worsening of the cardiac pump, and a more severe degree of mitral insufficiency (*p* < 0.05). Conversely, a higher rate of non-responders vs responders was found under ARNI and SGLT2-I therapy (*p* < 0.05) and showed the lowest expression of miRs (*p* < 0.05). Notably, miR-30 (β 0.340), 6MWT (β 0.168), ARNI (β 0.157), and SGLT2i therapy (β 0.245) correlated with significant improvements in LVEF (*p* < 0.05). In contrast, CRP values were inversely correlated (β-0.220) with significant improvements in the LVEF (*p* < 0.05). Intriguingly, miR-30 values (HR 2.7), ARNI therapy (HR 1.37), and the highest 6MWT values (HR 1.29) predicted the CRT responders via LBB pacing at 1 year of follow-up in T2DM patients (*p* < 0.05).

CRT could improve myocardial ventricular geometry and cardiac pump in a comparable proportion of diabetic and non-diabetic patients, along with a similar functional status amelioration (Sardu et al., 2014). These clinical effects could link to significant modulation of the miRs implied in cardiac remodeling in the CRT responders via over-expression of miR 26, miR 29, miR 30, miR 92, and miR 145 (Marfella et al., 2013; Sardu et al., 2016). In this setting, we could first confirm that LBB pacing is an alternative treatment for patients who cannot be treated by positioning a left ventricular lead into the CS (Vijayaraman et al., 2021; Chen et al., 2022). Second, LBB pacing is equal to CS pacing for achieving the best clinical outcomes in CRT patients (Vijayaraman et al., 2021; Chen et al., 2022). We confirmed this trend in our population of LBB-pacing patients; in particular, we positively correlated the miR-30 values to improving the cardiac pump. Furthermore, the highest miR-30 values increased by about 2.7 fold in the responders to LBB-pacing at the end of follow-up. The miRs investigated here could be implied in failing heart adaptive processes and modulated (overexpressed) in CRT responders.

The reversion of these adaptive cardiac processes is linked to LV reverse remodeling and the improvement of the cardiac pump, with consequent improvement of symptoms and clinical outcomes. Furthermore, we might speculate that the LBB pacing might regulate cardiac apoptosis, fibrosis, and angiogenesis by modulation of miRs (and expression of genes) implied in these cardiac remodeling processes. In this scenario, the miR-30 regulated myocardial hypertrophy by decreasing cystathionine-γ-lyase expression, hydrogen sulfide production, and reducing hypoxic cardiomyocyte injury in a murine model (Marfella et al., 2013; Sardu et al., 2016). In humans treated with CRT, the miR-30 could control cardiac angiogenesis and apoptosis, and it is significantly overexpressed in CRT responders (Marfella et al., 2013; Sardu et al., 2016).

Similarly, 6MWT (β 0.168), ARNI (β 0.157), and SGLT2i (β 0.245) linked to LV reverse remodeling at the end of follow-up (*p* < 0.05). In contrast, the inflammation (CRP, β −0.220) is inversely correlated to LV reverse remodeling at the end of follow-up. The inflammation is a well-known negative prognostic and diagnostic marker and trigger of HF in overall patients (Lappegård et al., 2015; Dick and Epelman, 2016) and particularly in those treated with CRT (Sardu et al., 2022). Indeed, over-inflammation could lead to cardiac remodeling via enhanced cardiac fibrosis and an increase of cardiac volumetry in CRT patients, then conditioning a worse prognosis (Marfella et al., 2013; Sardu et al., 2016; Sardu et al., 2022). Intriguingly, over-inflammation and the altered glycemic status could cause the modification of circulating proteins and ionic channels, which are implied in the entity of CRT response in T2DM patients (Gambardella et al., 2022). Indeed, this could cause the ryanodine receptor 1 (RyR1) glycation in circulating lymphocytes (Gambardella et al., 2022). The RyR1 glycation could predict CRT responsiveness via pathologic intracellular calcium leakage (Gambardella et al., 2022). In this setting, the 6MWT is a diagnostic, monitoring, and prognostic test of clinical outcomes in CRT patients (Gorcsan et al., 2008; Sardu et al., 2018a; Glikson et al., 2021). Indeed, HF patients with the highest values of 6MWT have the best cardiovascular performance and clinical outcomes (Gorcsan et al., 2008; Sardu et al., 2018a; Glikson et al., 2021). In this setting, we found that higher 6MWT values at baseline could link to significant improvement of the cardiac pump. The highest 6MWT values at baseline resulted in a 1.288-fold higher responder rate at the end of follow-up. Conversely, anti-HF drugs with anti-remodeling properties, such as ARNI (0.157) and SGLT2i (0.245), are linked to improvement of the cardiac pump. Furthermore, ARNI therapy increased the odds of responders post LBB pacing at 1 year of follow-up about 1.37 times. In this scenario, ARNI is the gold standard and first-step therapeutic approach to induce LV reverse remodeling with improvement of cardiac pump and the best clinical outcomes in HF patients (Gorcsan et al., 2008). In line with this result, CRT non-responders are divided into ARNI users vs non-ARNI users; the ARNI therapy could promote functional and clinical improvement by modulating the epigenetics of adverse molecular remodeling (Sardu et al., 2023). Indeed, the ARNI promoted a beneficial effect on cardiac dysfunction, dyssynchrony, and clinical status in non-responder CRT patients via the changes in miR plasma levels (Sardu et al., 2023). Similarly, SGLT2i are anti-remodeling drugs and a first-step therapeutic approach and gold standard therapy for HF patients (Gorcsan et al., 2008). Indeed, the SGLT2i showed cardiovascular and systemic protective effects via anti-inflammatory properties (Sardu et al., 2023; Donofrio et al., 2021) and inducing cardiac pathways implied in the modulation of cardiac systolic and diastolic function in humans beyond the glycemic control (Marfella et al., 2022a; Marfella et al., 2022b). Our data showed that SGLT2i therapy positively correlated with cardiac pump improvements at the end of follow-up (*p* < 0.05).

### Study limitations

Further studies using large-scale microarray profiling are needed to address the association between CRT via LBB pacing and miR changes in non-responding vs responding T2DM patients. We did not find a correlation between the causative or mechanistic relationship between LV reverse remodeling after CRTd and LBB pacing in the T2DM population. On the other hand, as seen in previous studies (Sardu et al., 2016; Vijayaraman et al., 2021), we demonstrated the relationship between clinical, molecular, and echocardiographic parameters of LBB-pacing responders with miR changes. Thus, the findings could indirectly support the favorable epigenetic effects of CRT via LBB pacing in HF patients with T2DM who cannot be treated by CRT via CS pacing. Again, the LBB pacing could lead to LV reverse remodeling and best clinical outcomes via a reduction of the inflammatory burden. This suggests a correlation between over-inflammation, miR expression, and LV reverse remodeling in HF patients treated with LBB pacing.

Conversely, here, we did not provide an animal model of chronic HF with T2DM or an *ex-vivo* model of cultured cardiomyocyte cells. Both these models could help, from one side, to test the effects of LBB pacing on inflammation/miR expression LV reverse remodeling and clinical outcomes. On the other side, they could be used to test the effects of specific treatment with mimic-miR on inflammation and the different remodeling cardiac processes in patients treated with LBB pacing. This new approach could be used in the clinic to improve outcomes.

In this context, the miRs could be markers and potential targets of cardiovascular diseases and HF (Zhu and Fan, 2011; Mahjoob et al., 2022; Macvanin et al., 2023). Thus, specific anti-remodeling therapies could be used to modify the expression of miRs (Brioschi et al., 2023) and lead to the best clinical outcomes. In this setting, specifically, the miR-30 is implied in LV remodeling, with ameliorative effects on the cardiac pump and best clinical outcomes (Melman et al., 2015). The miR-30 is induced by CRT (Melman et al., 2015). Further studies will be designed to address this hypothesis and identify novel molecular markers of reversed remodeling after LBB pacing. Finally, but not less relevant, we might consider cardiac magnetic resonance imaging (MRI) added to late gadolinium enhancement (LGE) imaging to investigate the cardiac fibrosis and reverse remodeling in LBB pacing patients at baseline and at the end of follow-up. Indeed, in patients without contraindications to MRI, the combination of LGE imaging and cine imaging would make MRI the modality of choice in assessing LV remodeling and cardiac pump function in HF patients (Cohn et al., 2000).

## Conclusion

LBB pacing is an alternative CRT technique that provides ventricular synchrony, induces LV reverse remodeling, and improves clinical symptoms in HF patients with LBB and T2DM. Notably, the patients who responded to LBB pacing evidenced a selective miR modulation, which was linked to significant improvement of cardiac pump in T2DM patients. Specifically, we highlighted miR-30 as the miR directly linked to improvement of cardiac pump and predictor of responders event at 1 year of follow-up.

## Data Availability

The raw data supporting the conclusions of this article will be made available by the authors, without undue reservation.
